# Elimination of a Mixture of Microplastics Using Conventional and Detergent-Assisted Coagulation

**DOI:** 10.3390/ma16114070

**Published:** 2023-05-30

**Authors:** Sabina Ziembowicz, Małgorzata Kida, Piotr Koszelnik

**Affiliations:** Department of Chemistry and Environmental Engineering, Faculty of Civil and Environmental Engineering and Architecture, Rzeszów University of Technology, AVE Powstańców Warszawy 6, 35-959 Rzeszów, Poland; mkida@prz.edu.pl (M.K.); pkoszel@prz.edu.pl (P.K.)

**Keywords:** microplastics, polyethylene, polyvinyl chloride, MPs mixture, coagulation process, surfactant, SDBS, LDIR analysis

## Abstract

The research described here investigated the suitability of coagulation process in the elimination of microplastics from tap water. The purpose of the study was to assess the effects of microplastic type (PE1, PE2, PE3, PVC1, PVC2, and PVC3), tap water pH (3, 5, 7, and 9), coagulant doses (0, 0.025, 0.05, 0.1 and 0.2 g/L), and microplastic concentration (0.05, 0.1, 0.15, and 0.2 g/L) on elimination efficiency with coagulation utilizing Al and Fe coagulants as well coagulation combined with a detergent (SDBS) addition. This work also explores the elimination of a mixture of two microplastics (PE and PVC) that are significant in terms of the environment. The effectiveness of conventional and detergent-assisted coagulation was calculated as a percentage. The fundamental characteristics of microplastics were also determined via LDIR analysis, and on the basis of these findings, particles that were more coagulation-prone were identified. The maximum reduction in MPs was achieved with tap water’s neutral pH and a coagulant dosage of 0.05 g/L. The addition of SDBS increased the loss of the plastic microparticles’ efficacy. A removal efficiency of greater than 95% (Al-coagulant) and 80% (Fe-coagulant) was achieved for each of the microplastics tested. The removal efficiency of the microplastic mixture with SDBS-assisted coagulation was obtained at a level of 95.92% (AlCl_3_·6H_2_O) and 98.9% (FeCl_3_·6H_2_O). After each coagulation procedure, the mean circularity and solidity of the unremoved particles increased. This confirmed that particles with irregular shapes are easier to completely remove.

## 1. Introduction

Considering their remarkable physical and chemical properties, inertness, wide-spread use, and reusability, plastics are a significant material in today’s society. However, despite their many advantages, plastics are linked to a wide range of environmental and human health problems due to their extremely slow natural biodegradation and release of toxic compounds [1,2]. Plastic pollution is a relatively new but rapidly spreading phenomenon. Over the past few decades, the annual production of plastic has increased over the world, increasing from about 1.5 million tons in 1950 to 368 million in 2019. By 2050, plastic production is expected to have increased to 2000 million tons [3].

Large plastics degrade through contact with the environment due to physical, chemical, and biological processes, including mechanical, biochemical, thermal, UV, and biological deterioration, as well as mechanical forces and turbulence [4,5,6,7]. Plastic fragmentation leads to the formation of smaller particles, including microplastics (MPs) [8]; microplastics are defined as plastic particles with sizes in the range of 1 to 5000 µm [9,10,11]. MPs are classified as primary when they are produced in small sizes and secondary when they are formed in the environment as a result of the slow decay of large plastic parts. Due to their versatility and widespread application, MPs are present in the environment. The presence of microplastics has been confirmed in soil [12,13,14], subsurface systems [15,16,17], groundwater [18,19,20], the atmosphere [21,22], rivers [23,24,25,26], and marine environments [27,28,29].

Given that freshwater is seen as a substantial source of MPs in the oceans, attention to microplastics in freshwater ecosystems needs to be increased. Freshwater immediately affects people’s life because most freshwater rivers provide people with their daily drinking water. Freshwater resources are fed into drinking water treatment plants (DWTPs) to supply people with drinking water. However, DWTPs are also not able to completely remove MPs; consequently, a large number of MPs were observed drinking treated water. [30,31,32,33]. For example, Tong et al. (2020) [34] reported that adults are prone to ingest 660 MPs per day with tap water. Based on the identification of MPs in treated water, many morphologies have been observed, including fibers, pieces, spheres, and films [30,31,32]. It is challenging to study microplastic removal because of their heterogeneous nature. Although MPs are described as synthetic polymers that are smaller than 5 mm in length in the literature, there is not a single definition that is universally acceptable. Plastics represent a broad variety of materials, each with distinct physical properties and chemical compositions. However, of the total amount of plastic manufactured worldwide, around 90% belongs to one of five groups: PE, PP, PVC, PS, and PET [35]. It has been shown that microplastics are stable in water due to their chemical stability, even over thousands of years. With the gradual entry of microplastics into the waters, serious environmental and health problems have been induced. Microplastic particles have a large specific surface area and strong hydrophobic characteristics, making them good toxic pollutant carriers. Due to their small particle size, microplastics can easily be mistaken for food by aquatic species. In addition, when additives are leached from microplastics during their disintegration, they pose a major threat as hazardous compounds. These included bisphenol A and phthalates, which are added as plasticizers to plastic [36,37,38,39,40].

An essential step in water treatment facilities is the coagulation process, which is also connected to the quantity of MPs entering the aquatic environment. DWTPs are the link between surface water and tap water. The coagulation/flocculation process can play an important role in the removal of MPs [41], although the removal rate varies depending on a number of parameters, including the type and dosage of the coagulant.

In the coagulation/flocculation process, iron and aluminum salts are usually applied as coagulants. Ariza-Tarazona et al. (2019) [42] studied the removal of polyethylene microplastics using iron and aluminum salt coagulants. The coagulation processes were carried out at different concentrations of Al^3+^ and Fe^3+^ ions, and the results showed that the Al coagulant is more effective than Fe is. For example, Ma et al. (2019a) [43] investigated the removal of pristine microparticles of PE using ferric chloride as a coagulant and observed that 90.91 ± 1.01% of MPs were removed by a 2 mM coagulant (540 mg/L of the coagulant) with the addition of 15 mg/L of polyacrylamide. Furthermore, Zhang et al. (2021b) [44] observed a removal efficiency of 91.45% for polyethylene terephthalate (PET) MPs using 200 mg/L of polyaluminum chloride and 100 mg/L of PAM. Ma et al. [43] studied the coagulation of PE microplastics using AlCl_3_·6H_2_O and FeCl_3_·6H_2_O as coagulants. The authors determined that the smaller the particles, the higher the removal yield. The highest efficiency achieved was less than 40% for PE particles smaller than 500 µm, but this efficiency increased to over 60% after the addition of anionic polyacrylamide (15 mg/L). Skaf et al. [45] also observed a positive effect indicated by the presence of an anionic surfactant. However, Xia et al. [46] observed a negative effect of non-ionic surfactants. The authors determined that the presence of nonionic surfactants hinders the fusion of microparticles with aluminum flocs during the coagulation process.

As stated in scientific reports, coagulation specifically affects the removal of microplastics. However, there are different forms of microplastics in different bodies of water, and various coagulants have different results. Furthermore, the precise mechanism is unclear. Coagulation is one of the most crucial technologies for removing MPs in DWTPs; therefore, it is urgently necessary to better the understanding of the mechanisms and factors that influence MP elimination. However, to the knowledge of the authors, no studies have examined the effect of coagulation on microplastic mixture elimination. The novelty of our research is in the use of coagulation to remove the mixtures of different kinds of microplastics. Preliminary research results, which were obtained by the authors of the paper in which they were reported were published by Ziembowicz et al. (2023) [47]. However, these studies concerned only one type of PE and PVC. In this article, three types of polyethylene and three types of polyvinyl chloride, as well as their mixtures, were analyzed. Existing studies have tested the effectiveness of eliminating one type of microplastics [42,43,44,45,46]. Therefore, this study aims to compare the use of AlCl_3_·6H_2_O and FeCl_3_·6H_2_O as coagulants to remove a significant plastic element of MPs (polyethylene, and polyvinyl chloride). The utilitarian objectives were to study the possibilities of improving the coagulation process by adding a surfactant. Moreover, analyses using LDIR (Laser Direct Infrared) were used to evaluate the obtained research results.

## 2. Materials and Methods

### 2.1. Chemicals and Materials

AlCl_3_·6H_2_O and FeCl_3_·6H_2_O as coagulants, anionic surfactant sodium dodecyl benzenesulfonate (SDBS), hydrochloric acid, sodium hydroxide, humic acid, sodium chloride and ethanol were obtained from Sigma-Aldrich (Saint Louis, MO, USA). The tap water used in the study was tap water from Rzeszow. The water parameters are presented in Table 1. The filters were obtained from Whatman glass microfiber-(GF/A; diameter—47 mm, pore size—1.6 µm).

Three commercially available types of PE and three PVC microplastics were purchased from Sigma-Aldrich (USA). Information on the microplastics tested is presented in Table 2.

### 2.2. Coagulation Experiments

A 1 L beaker with a Stuart flocculator with six rotators was used for coagulation experiments. The amount of water in each sample was 500 mL. By adding HCl or NaOH, the pH of tap water was kept at 3, 5, 7, and 9. The amounts of individual microplastics made of PE and PVC were 0.05, 0.1, 0.15, and 0.2 g/L. The microplastic mixture was used in the following combinations: MIX1 (0.025 g PE1 + 0.025 g PVC1), MIX2 (0.025 g PE2 + 0.025 g PVC2), and MIX3 (0.025 g PE3 + 0.025 g PVC3). Weighed microplastics were added to water and mixed. In this investigation, Al- and Fe-based coagulants were utilized at doses of 0, 0.025, 0.05, 0.1, and 0.2 g/L. The conventional coagulation method was modified by adding 20 mg/L of SDBS. The mixing rate was kept at 300 rpm/min for 1 min, then 50 rpm/min for 15 min, with a subsequent sedimentation of 45 min. The supernatant was obtained following sedimentation in order to determine the effectiveness of the removal of microplastics. A minimum of three repetitions of each experiment were performed.

### 2.3. Measurement of Microplastics

The collected supernatants were used for the measurement of the microplastics remaining after coagulation. All supernatants were filtered through pre-weighed Whatman filters. The residuals flocs were eliminated using a HCl solution. The MP-containing filters were dried for 18 h at 60 °C. Subsequently, the MP-containing membranes were weighted after they cooled to room temperature. The total removal efficiency of microplastics removal was calculated using the following formula:

(1) $$E=\frac{m_{0}-m_{t}}{m_{0}}\;\times \;100\%$$

where E is the removal efficiency of microplastics during coagulation (%), *m*_0_ is the mass of microplastics in the solution at the beginning of the coagulation process (g), and *m_t_* is the mass of microplastics in the supernatant after the coagulation process (g).

### 2.4. Characteristics of Microplastics

The microplastic samples were deposited on infrared reflective glass slides (Kevley Technologies, Chesterland, OH, USA). The samples were analyzed via transflection using automated LDIR imaging with Quantum Cascade Laser (Agilent 8700). Diameter range and mean diameter, area, perimeter, circularity, and solidity were determined. A proprietary quantum cascade laser (QCL) was used as a light source operating at high speeds, with an elevated wavelength accuracy (spectral resolution of 8 cm^−1^) and fast-scanning optics using a full spectrum in the mid-IR range.

## 3. Results and Discussion

### 3.1. Performance of Coagulation to Remove PE and PVC—Impact of Coagulant Dose and Microplastics Type

**Figure 1:**
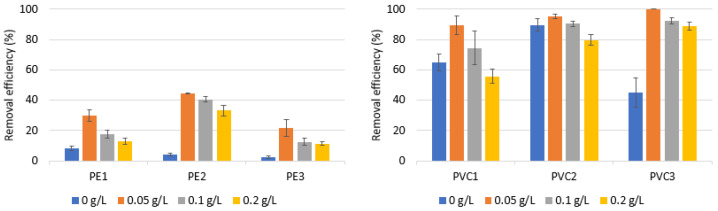
Removal efficiency of microparticles at pH 7.0 with different coagulant doses in the presence of AlCl_3_·6H_2_O. Other experimental parameters: concentration of MPs, 0.1 g/L; water volume, 500 mL.

Figure 1 demonstrates the removal efficiency of PE and PVC with various dosages of coagulants. AlCl_3_·6H_2_O was chosen to determine how the coagulant dose affected efficiency. The coagulant doses utilized were 0, 0.05, 0.1, and 0.2 g/L, which corresponded to Al concentrations of 0, 0.21, 0.42, and 0.84 mM/L, respectively.

The removal effectiveness of PE tested in the absence of a coagulant was between 2.35% and 8.44%, while the removal efficiency of the PVC tested was between 44.98% and 89.73%. This fact is related to various densities (the PE and PVC density is 0.88–0.96 and 1.38 g/mL, respectively). For the same reason, significant differences in the removal efficiency of PE and PVC during the coagulation process were observed. The presence of an Al coagulant increased the removal efficiency of the tested particles. As shown in Figure 1, the dose of 0.05 g/L was observed to have the best removal effectiveness for both PE and PVC. Increasing the dose of the coagulant did not only improve the efficiency of the process, but also resulted in a lower efficiency of the removal of the microplastics tested. This may be explained with the observation that flocs tend to loosen and break easily when the coagulant dose is too high [48,49]. In addition, higher doses of the coagulant caused difficulties in the filtration process and an increased consumption of hydrochloric acid to remove flocs from the filter. The removal efficiency of the PE microparticles tested during coagulation was in the range of 11.36–44.30%. A similar observation was found by Ma et al. [43] who found that the removal efficiency of PE MPs was below 40% in the presence of an Al-based coagulant. The examined PVC microparticles had a removal effectiveness of between 55.63% and 100% during coagulation. A statistically significant difference was found between the removal efficiency and the coagulant dose, for PVC1 (*p* < α, *p* = 0.0345), PVC2 (*p* < α, *p* = 0.0249), PVC3 (*p* < α, *p* = 0.0176), PE1 (*p* < α, *p* = 0.0156), PE2 (*p* < α, *p* = 0.0156) and PE3 (*p* < α, *p* = 0.0216).

The type of microplastic removed also affects the effectiveness of the coagulation process. Different removal efficiencies were found for PVC1 and PVC2, although they had the same particle size range (Table 2). PVC2, a high-molecular-weight polymer, showed a greater degree of removal. In turn, as a result of coagulation, the highest efficiency was observed for the largest-size particles of PVC3. Unlike PVC, where particles spontaneously sank to the bottom, the primary process for removing microplastics from polyethylene was based on coagulation. For polyethylene microparticles, the highest efficiency (44.30%) was observed for PE2 (20–241 µm particles size). A statistically significant difference was found between the removal efficiency and the type of MP for a coagulant dose of 0.05 g/L (*p* < α, *p* = 0.0056).

### 3.2. Performance of Coagulation to Remove PE and PVC—Impact of Coagulant Type

**Figure 2:**
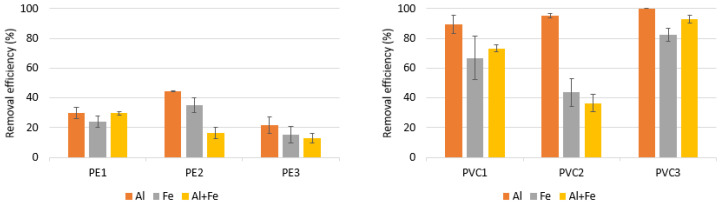
Removal efficiency of microparticles using various coagulants at pH 7.0. Other experimental parameters: concentration of MPs, 0.1 g/L; water volume, 500 mL; coagulant dosage, 0.05 g/L (Al + Fe: 0.025 g/L of AlCl_3_·6H_2_O + 0.025 g/L of FeCl_3_·6H_2_O).

Figure 2 shows the results for the coagulation process depending on the coagulants used. Coagulation processes using of an aluminum coagulant, iron coagulant, and a mixture of these two coagulants in equal amounts were compared (0.025 g/L of FeCl_3_·6H_2_O and 0.025 g/L of FeCl_3_·6H_2_O). The optimal coagulant dose was used; that is, FeCl_3_·6H_2_O at a dose of 0.05 g/L corresponding to Fe concentrations of 0.18 mM/L. The average removal effectiveness for PE and PVC microplastics was found to be lower when using the Fe coagulant. Aluminum coagulation is reported to be more efficient. The reason might be the different character of the sludge. Aluminum sludge contains more water than iron sludge contains, which is a potential reason why microplastics are better removed. The reason might be the different character of the sludge. Aluminum sludge contains more water than iron sludge contains, and occurs not only via adsorption on the surface of the sludge, but also via the capture of the water volume, and that is a potential reason why microplastics are better removed [8].

However, after using two coagulants at the same time in the case of removing PE1, PVC1 and PVC3, better efficiency was observed with the use of the mixture of two coagulants than with the use of iron salts alone as the coagulant. Furthermore, PE1 had the same elimination efficiency both when using a mixture of coagulants and the coagulant AlCl_3_·6H_2_O alone (Figure 2). This is particularly important in the case of the required dose of Al and Fe, which exceeds the permissible concentration in water. For actual drinking water treatment, previous research indicated that the content of Al- and Fe-based coagulants (estimated as 0.74 mM of Al and 0.36 mM of Fe) should be less than 0.02 g/L [43,50,51,52].

### 3.3. Performance of Coagulation to Remove PE and PVC—Impact of pH and MP Initial Concentration

The effectiveness of coagulation for the removal of MPs is greatly influenced by the pH of water and the initial concentration of pollutants. The pH level affects the hydrolysis of the coagulant and the effectiveness of the coagulation [43,48]. The removal effectiveness of PE and PVC microplastics at various starting pH values is shown in Table 3. To test the effectiveness of MP removal, experiments were run at pH levels 3, 5, 7, and 9. According to the findings, both Al- and Fe-based coagulants removed PE and PVC with the maximum effectiveness under neutral conditions (pH 7). Similar conclusions have been obtained in other studies [53,54,55]. Shen et al. [55] investigated the removal performance of microplastics in wastewater via electrocoagulation. The highest degree of elimination was observed in the pH range of 5–7.2. The lower and higher pH resulted in a significant reduction in the coagulation efficiency. All microplastics are negatively charged while in neutral conditions, which makes them more conductive when paired with positively charged flocs to remove microplastics from tap water [54]. The coagulation procedure can be applied to nearly all water-containing microplastics because tap water’s ideal pH range is 6.5 to 9.5, negating the need to add additional chemicals to modify the pH [52].

**Figure 3:**
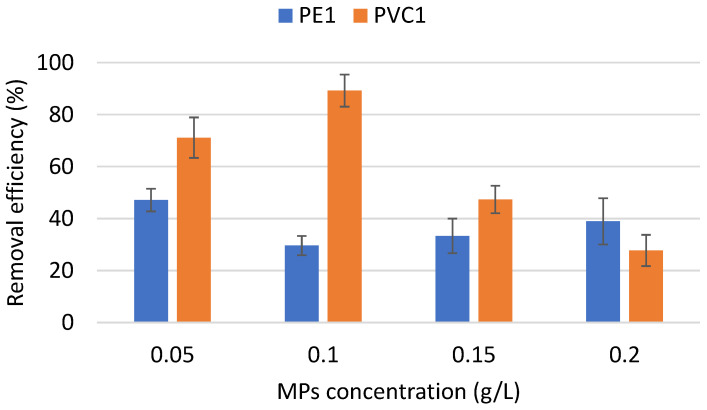
Removal efficiency of PE1 and PVC1 particles under various initial MP concentrations. Other experimental parameters: pH, 7.0; coagulant dosage, 0.05 g/L, water volume, 500 mL.

The concentration of MPs in the water is also an important factor that affects the removal efficiency of coagulation. Figure 3 illustrates how the initial concentration of PE1 and PVC1 affects how effectively the coagulation process works. In tap water containing PE1 and PVC1 in concentrations of 0.05, 0.1, 0.15, and 0.2 g/L, a coagulation procedure was performed using Al salts. The initial concentration of microplastics had an impact on how well PE1 and PVC1 could be removed. The level of PE1 removal was the highest at a concentration of 0.05 g/L. Doubling the initial amount of PE1 resulted in a 17.5% decrease in coagulation efficiency. When the concentration was further increased, there was a slight increase in efficiency compared to that with 0.1 g/L, but there was still lower efficiency compared to that with 0.05 g/L. The best PVC1 removal efficiency was reported at a concentration of 0.1 g/L. A lower coagulation efficiency was observed both for a lower initial concentration and for a higher concentration.

### 3.4. Performance of Coagulation to Remove PE and PVC—Impact of SDBS Addition

**Figure 4:**
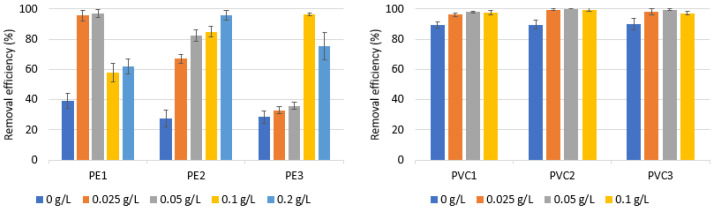
The effect of adding SDBS (20 mg/L) on the effectiveness of removing PE and PVC at different coagulant doses at pH 7.0 in the presence of AlCl_3_·6H_2_O. Other experimental parameters: concentration of the different PE particles, 0.1 g/L; water volume, 500 mL.

**Figure 5:**
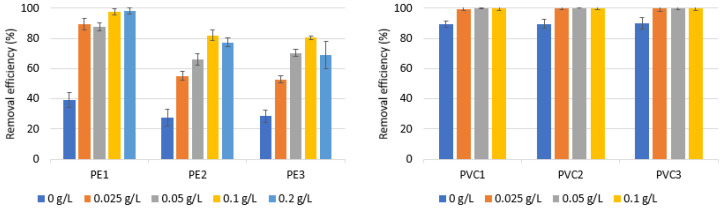
The effect of adding SDBS (20 mg/L) on the effectiveness of removing PE and PVC at different coagulant doses at pH 7.0 in the presence of FeCl_3_·6H_2_O. Other experimental parameters: concentration of the different PE particles, 0.1 g/L; water volume, 500 mL.

Figure 4 and Figure 5 display the impact of SDBS addition (20 mg/L) on the effectiveness of PE and PVC removal. Sodium dodecylbenzene sulfonate is an anionic surfactant with properties of detergency, moistening, foaming, emulsification, and dispersity [56]. The addition of a surfactant results is the average concentration of the surfactant in wastewater [55].

Analyzing the results in Figure 4 and Figure 5, it can be concluded that removing PE and PVC from the coagulation of an Al salt and Fe salt was greatly improved with the addition of SDBS. A removal efficiency of above 95% (Al-coagulant) and 80% was achieved (Fe coagulant for each of the types of microplastics). It is also possible to reduce the dose of the coagulant while maintaining a high efficiency of removal of PE and PVC by adding SDBS. For example, comparing a dose of the Al coagulant of 0.025 g/L + SDBS addition with the dose of the coagulant of 0.05 g/L for PE1, PE2 and PE3, the removal efficiency was higher: 66.02, 22.79 and 11.39% higher, respectively. A statistically significant difference was found between the removal efficiency andthe coagulant dose, for PVC2 (*p* < α, *p* = 0.0412), PVC3 (*p* < α, *p* = 0.0176), PE1 (*p* < α, *p* = 0.0136), PE2 (*p* < α, *p* = 0.0111) and PE3 (*p* < α, *p* = 0.0102), while a statistically significant difference was not found for PVC1. Additionally, a statistically significant difference was found between the removal efficiency andthe kind of MP for a coagulant dose of 0.025 g/L (*p* < α, *p* = 0.0087).

In the absence of a coagulant, PE had an elimination efficiency of between 27.44 and 39.41% and that of PVC was between 89.35 and 90.12%. MPs are hydrophobic organic pollutants but, when surfactants are added, their physical and chemical characteristics change. Adsorption of coexisting surfactants into MPs can alter their hydrodynamic properties [46]. In a study by Shen et al. in 2022 [55], a SDBS was mainly employed to make sure that PE microplastics were entirely dispersed in water. However, the research results show a positive effect of the presence of SDBS on microplastic sedimentation.

### 3.5. Performance of Coagulation to Remove Mixture of PE and PVC

**Figure 6:**
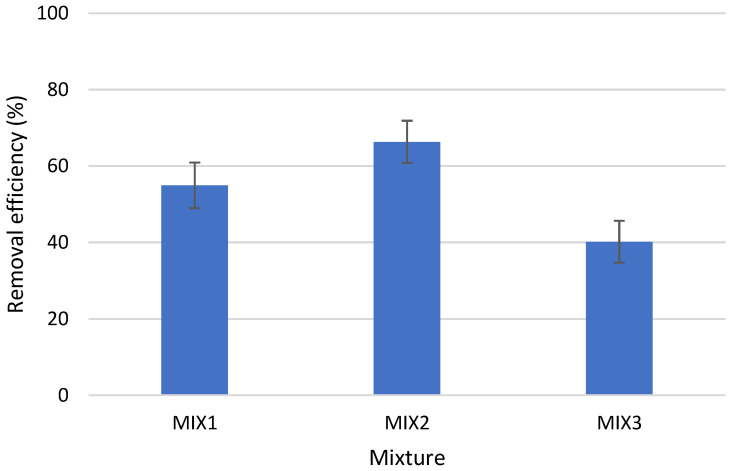
Removal efficiency of PE and PVC mixture particles in the presence of AlCl_3_·6H_2_O. Other experimental parameters: concentration of the MP particles, 0.1 g/L (0.025 g PE + 0.025 g PVC); coagulant dosage, 0.05 g/L; water volume, 500 mL.

The studies also attempted to use coagulation in tap water that contained a variety of mixture of PE and PVC in equal amounts by weight. Because water and wastewater include a mixture of microplastics, not a single material, this is a crucial factor. Therefore, studies on the elimination of pollutants should focus on the entire collection of pollutants rather than specific chemicals. The established procedure and its parameters frequently work well to eliminate only this one chemical. Industrial-scale wastewater and water treatment applications require a universal technique that can eliminate a wide range of contaminants. For MIX1, MIX2, and MIX3, the effectiveness of microplastic elimination via coagulation with the application of the Al coagulant was found to be at a level of 54.92, 66.33, and 40.17%, respectively (Figure 6). A statistically significant difference was found for the removal efficiency in the mixed kind (*p* < α, *p* = 0.0273). The highest efficiency was observed for MIX2, which consists of PE2 and PVC2, molecules with the highest degree of elimination in a one-component system (individual microplastics) (Figure 1).

The unremoved PE and PVC particles were evaluated to figure out which particles coagulated less after the operation. The following parameters of the molecules were determined as percentages: diameter range, mean diameter, area, perimeter, circularity, and solidity. The results are shown in Table 4 of the report. The degree to which a particle resembles a circle, taking into consideration the smoothness of the perimeter, is known as its circularity. A perfect circle has a circularity rating of 1.0. A particle’s solidity is determined by comparing its actual surface area to the surface area created by a thread wrapped around it. Analyzing the information in Table 2, it is possible to observe a change in each of the evaluated parameters. The percentage of polyethylene microparticles after coagulation for MIX1 and MIX2 increased significantly, while that for MIX3 decreased from 50 to 44%. The percentage of PVC3 in turn increased by 6%. The lowest coagulation efficiency with an Al coagulant was also observed for MIX3.

The detection of larger particles after coagulation processes indicates that during coagulation, smaller and larger particles combine to form flocs. On the other hand, the presence of particles that are smaller than the original ones support the idea that throughout the coagulation process, the particles are subjected to various stresses and broken up into smaller ones. The rise in mean diameter is correlated with an increase in particle area and perimeter. The shape of the particles that were not removed during the coagulation process was more regular and spherical. This is evidenced by the increase in parameters such as mean circularity and solidity (Table 4).

**Figure 7:**
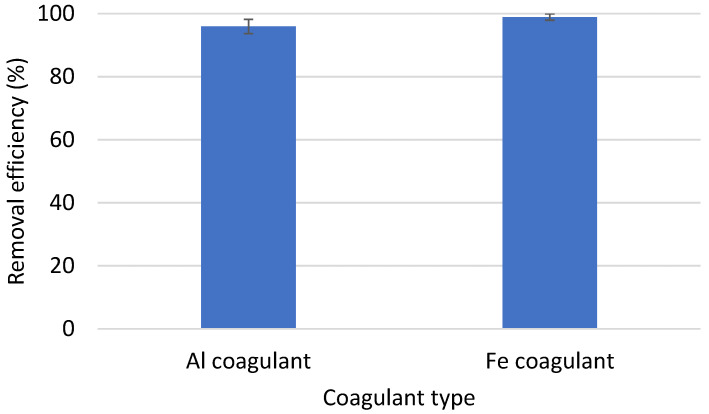
Removal efficiency of PE and PVC mixture particles in the presence SDBS. Other experimental parameters: concentration of the MP particles, 0.1 g/L (0.025 g PE1 + 0.025 g PVC1); coagulant dosage, 0.05 g/L; water volume, 500 mL.

The MIX 1 mixture was selected for further studies on the degree of elimination in coagulation with the addition of SDBS. The usefulness of SDBS-assisted coagulation was checked for both the use of AlCl_3_·6H_2_O and FeCl_3_·6H_2_O. The results of the coagulation efficiency are presented in Figure 7. Al and Fe coagulants were used with respective efficiencies of 95.92% and 98.9% for the removal of microplastics during coagulation. This means that the addition of the surfactant increased the elimination of the tested microplastic mixture from 54.92 to 95.92% in the case of the aluminum-based coagulant. Furthermore, the use of coagulation with the use of iron coagulant and SDBS showed even higher elimination, up to 98.9%, of microplastics.

The unremoved PE1 and PVC1 particles were also examined to discover which particles are more susceptible to coagulation. Table 5 shows the percentages of various microplastics before and after the coagulation operations and the characteristics of the microplastic particles. When AlCl_3_·6H_2_O was used as a coagulant, the percentage of PE remaining in the water increased from 53 to 90.3%; however, when FeCl_3_·6H_2_O was used, it increased to 79.6%. The opposite trend was shown in the case of PVC. This indicates that the PVC microplastic is more likely to coagulate with Al salt, while PE is more likely to be removed by an Fe coagulant. The mean circularity and solidity of the unremoved particles were higher after each coagulation process. This shows that irregularly shaped particles are easier to remove. Compared to that of spherically shaped particles, the surface area of irregular MPs is larger. Because the surface area of irregular MPs is larger, the adsorption of the MPs by a coagulant is supposed to be more pronounced compared with spherical-shape MPs [57].

## 4. Conclusions

This study systematically explored the removal performance of six microplastic materials (three types of PE and three types of PVC, individually or in mixtures) in tap water via conventional coagulation and detergent-assisted coagulation. The effects of coagulant (AlCl_3_·6H_2_O and FeCl_3_·6H_2_O), pH value of tap water, the doses of coagulant and the microplastic concentration on the process were studied. The findings suggested that using coagulation technology is an effective method for removing microplastics from tap water. Compared to the process with the Fe coagulant, the Al coagulant had a better removal effect on all microplastics. When the initial pH of tap water was neutral (pH 7), the removal effect of microplastics reached the highest with the final removal rate of 29.62% for PE1, 44.3% for PE2, 28.32% for PE3, 89.24% for PVC1, 95.48% for PVC2 and 100% for PVC3. The optimal dose of the coagulant should also be determined, because too small and too large a dose negatively affects the effectiveness of coagulation. The highest removal of each MP examined occurred at the coagulant dosage of 0.05 g/L. Optimal coagulation conditions were applied to remove the analyzed materials in three different mixtures, and the efficiency obtained was in the range of 40–66%. The second stage of the research consisted of the use of coagulation assisted by the addition of the SDBS surfactant to tap water before the coagulation process. The performance of removal of PE and PVC microparticles in both Al salt and Fe salt coagulation was greatly improved by the addition of SDBS. A removal efficiency of above 95% (Al coagulant) and 80% (Fe coagulant) was achieved for each of the types of microplastics tested. The efficiency of the removal of the microplastic mixture (PE1 + PVC1) with SDBS-assisted coagulation with AlCl_3_·6H_2_O and FeCl_3_·6H_2_O was obtained at the level of 95.92% and 98.9%, respectively

Coagulation technology has been successfully applied in water treatment for the elimination of microplastics. This is confirmed by the results obtained in this publication and the research conducted by other research teams [29,30,32,43,45,48,58,59,60,61]. The cohabitation of various types of microplastics and other contaminants in the water make it more difficult to effectively remove them. It is suggested that we further study the effect of developed technology on the effectiveness of eliminating other types of microplastics with different compositions, shapes, sizes, and levels of degradation (primary and secondary MPs). The correlation between operation cost and microplastic removal efficiency of using coagulation technology should be considered.

## Figures and Tables

**Table 1:** The selected parameters of tap water.

Parameters	Unit	Average Value
Color	mg Pt/L	<5
Turbidity	NTU	<0.20
pH	-	7.70
Conductivity	µS/cm	647
NO_3_^−^	mg/L	6.3
NO_2_^−^	mg/L	<0.05
Cl^−^	mg/L	38
SO_4_^2−^	mg/L	36
Total organic carbon	mg/L	1.50
Total hardness	mg CaCO_3_/L	260

**Table 2:** Characteristics of microplastics.

MPs	Characteristics
PE1	Ultra-high molecular weight, surface-modified, powder, 15–97 μm particle size *
PE2	Ultra-high molecular weight, surface-modified, powder, 20–241 μm particle size *
PE3	Ultra-high molecular weight, average Mw of 3,000,000–6,000,000, 69–250 μm particle size *
PVC1	Low molecular weight, 50–162 μm particle size *
PVC2	High molecular weight, 51–165 μm particle size *
PVC3	Average Mw of ~233,000, average Mn of ~99,000, 19–331 μm particle size *

* Own LDIR analysis.

**Table 3:** Effect of solution pH on PE and PVC removal efficiency.

Removal Efficiency (%)						
MPs	PE1		PE2		PE3	
Coagulant	Al	Fe	Al	Fe	Al	Fe
pH = 3	4.2 ± 0.6	5.81 ± 0.9	20.2 ± 4.2	9.6 ± 3.1	20.2 ± 3.9	8.2 ± 2.1
pH = 5	19.5 ± 2.7	5.88 ± 0.6	40.9 ± 2.5	10.2 ± 3	25.3 ± 4	6.9 ± 3
pH = 7	29.6 ± 3.7	18.31 ± 6.8	44.3 ± 4	31.7 ± 5.1	28.3 ± 4.2	15.4 ± 2.2
pH = 9	14.9 ± 1.9	9.0 ± 1	19 ± 2	23 ± 4	20.3 ± 5	12.1 ± 2.3
**Removal Efficiency (%)**						
**MPs**	**PVC1**		**PVC2**		**PVC3**	
**Coagulant**	**Al**	**Fe**	**Al**	**Fe**	**Al**	**Fe**
pH = 3	51.8 ± 7.8	38.4 ± 5.7	52 ± 3.8	52.62 ± 4.9	42 ± 5.5	31.72 ± 3.2
pH = 5	49.1 ± 8.9	41.8 ± 10	63 ± 4	35.54 ± 4.4	65 ± 7.2	80.63 ± 3.1
pH = 7	89.2 ± 6.2	66.8 ± 14	95.5 ± 4	43.72 ± 5.2	100 ± 2	82.42 ± 3.4
pH = 9	46.6 ± 3.5	39 ± 3.5	63 ± 5	40 ± 7.1	80 ± 7.1	63 ± 3.2

**Table 4:** Characteristics of microplastics before and after the coagulation process.

MIX	MPs	Percentages (%)	Diameter Range (µm)	Mean Diameter (µm)	Mean Area (µm²)	Mean Perimeter (µm)	Mean Circularity	Mean Solidity
MIX1	PE1 before	53	15–97	51.83	3126.68	225.6	0.65	0.900
	PE1 after	86	14–201	53.21	2962.05	221.91	0.66	0.904
	PVC1 before	47	50–162	136.79	17,993.89	653.06	0.53	0.86
	PVC1 after	14	23–416	165.91	26,350.19	769.83	0.76	0.95
MIX2	PE2 before	58	20–241	124.92	10,021.49	379.57	0.68	0.93
	PE2 after	91	31–415	156.35	22,810.99	628.61	0.78	0.97
	PVC2 before	42	51–165	120.2	45,202.94	968.75	0.56	0.85
	PVC2 after	9	103–422	216.11	41,527.94	925.45	0.57	0.89
MIX3	PE3 before	50	69–250	156.72	14,735.44	434.29	0.68	0.92
	PE3 after	44	18–484	191.79	24,536.79	620.73	0.75	0.95
	PVC3 before	50	19–331	179.72	31,508.76	745.49	0.59	0.89
	PVC3 after	56	25–497	225.5	46,824.47	958.53	0.67	0.93

**Table 5:** Characteristics of microplastics before and after the coagulation process with the addition of SDBS.

	Particle Parameters						
Coagulation Process	Percentages (Remaining in the Water)	Diameter Range (µm)	Mean Diameter (µm)	Mean Area (µm²)	Mean Perimeter (µm)	Mean Circularity	Mean Solidity
	**PE1**						
Before	53%	15–97	51.83	3127	225.6	0.65	0.900
AlCl_3_·6H_2_O	90.3%	14–253	43.45	2316	165.95	0.75	0.94
FeCl_3_·6H_2_O	79.6%	24–187	73.34	4297	256.77	0.74	0.95
	**PVC1**						
Before	47%	50–162	136.79	17,994	653.06	0.53	0.86
AlCl_3_·6H_2_O	9.7%	25–238	123.88	13,074	476.64	0.71	0.94
FeCl_3_·6H_2_O	20.4%	81–258	137.95	13,899	492.41	0.72	0.95

## Data Availability

The data that support the findings of this study are available from the corresponding author, upon reasonable request.
